# Fungal Infections Other Than Invasive Aspergillosis in COVID-19 Patients

**DOI:** 10.3390/jof8010058

**Published:** 2022-01-06

**Authors:** Kerri Basile, Catriona Halliday, Jen Kok, Sharon C-A. Chen

**Affiliations:** 1 Centre for Infectious Diseases and Microbiology Laboratory Services, NSW Health Pathology—Institute of Clinical Pathology and Medical Research, Westmead, NSW 2145, Australia; kerri.basile@health.nsw.gov.au (K.B.); Catriona.Halliday@health.nsw.gov.au (C.H.); jen.kok@health.nsw.gov.au (J.K.); 2 Centre for Infectious Diseases and Microbiology—Public Health, Westmead Hospital, Westmead, NSW 2145, Australia; 3 Sydney Institute for Infectious Diseases, University of Sydney, Sydney, NSW 2006, Australia

**Keywords:** COVID-19, SARS-CoV-2, fungal infections, non-*Aspergillus* fungi

## Abstract

Invasive fungal disease (IFD) associated with Coronavirus Disease 2019 (COVID-19) has focussed predominantly on invasive pulmonary aspergillosis. However, increasingly emergent are non-*Aspergillus* fungal infections including candidiasis, mucormycosis, pneumocystosis, cryptococcosis, and endemic mycoses. These infections are associated with poor outcomes, and their management is challenged by delayed diagnosis due to similarities of presentation to aspergillosis or to non-specific features in already critically ill patients. There has been a variability in the incidence of different IFDs often related to heterogeneity in patient populations, diagnostic protocols, and definitions used to classify IFD. Here, we summarise and address knowledge gaps related to the epidemiology, risks, diagnosis, and management of COVID-19-associated fungal infections other than aspergillosis.

## 1. Introduction

Severe acute respiratory syndrome coronavirus-2 (SARS-CoV-2), the virus responsible for Coronavirus Disease 2019 (COVID-19), was first identified in December 2019 in Wuhan, China and declared a pandemic by the World Health Organization (WHO) in March 2020 [1]. Despite the rapid development of pathogen-specific therapies and vaccinations beginning with emergency use authorisation of the Pfizer-BioNTech (Pfizer Inc., New York, NY, USA) COVID-19 mRNA BNT162b2 vaccine in December 2020 [2], there remains ongoing worldwide transmission of SARS-CoV-2 accelerated by the emergence of WHO designated variants of concern (VOC) [3] strains, which have fuelled increased risks to public health. The clinical spectrum of COVID-19 ranges from asymptomatic infection to severe respiratory illness and multiorgan failure; extrapulmonary disease may also occur [4,5]. Furthermore, morbidity is compounded in that COVID-19 per se, as well as the therapeutic agents used for its treatment, predisposes to other infections including fungal infections, which may co-exist or follow COVID-19 [6]. Uncommon early in the pandemic [6], fungal coinfections are now increasingly reported [7,8] of which the most well characterized is invasive aspergillosis (IA) [9,10,11,12,13]. 

Opportunistic invasive fungal disease (IFD) in the setting of severe respiratory viral illness is not novel, being well described in the context of severe influenza, parainfluenza, and respiratory syncytial virus infections, and now, COVID-19 [10,14,15,16,17,18]. Whilst COVID-19-associated pulmonary aspergillosis (CAPA) [13] was the first IFD to be reported and is the most well-established clinical entity, reports of fungal coinfections due to yeasts and non-*Aspergillus* filamentous fungi have increased [19,20,21,22]. The recognition of these IFDs is essential for early and targeted treatment, as antifungal drug choices differ from those for aspergillosis. In turn, this hinges upon the rapid, accurate identification of the etiological pathogen by laboratory tests given the common symptoms (fever, cough, and dyspnoea) [23] and radiological findings e.g., ground glass opacities [23,24] in CAPA and non-CAPA IFD. However, laboratory diagnosis is challenged by reticence in performing procedures such as bronchoscopies and induced sputum collection to prevent the nosocomial transmission of COVID-19.

Despite improvements in the understanding of CAPA and other COVID-19-associated fungal infections, knowledge gaps related to diagnosis, management, and prevention remain. We first briefly describe the pathophysiology of COVID-19 lung disease and the risks for IFD in COVID-19 patients (Section 2 and Section 3). Then, we review COVID-19-associated IFD caused by fungi other than *Aspergillus* spp. focussing on the epidemiology, diagnostic, and management approaches (Section 4). Finally, we summarise the more pertinent microbiological and imaging findings that may assist the diagnosis of non-*Aspergillus* IFD in COVID-19 patients (Section 5).

## 2. Pathophysiology of COVID-19 Lung Disease

There are several pathophysiological mechanisms by which SARS-CoV-2 as well as its treatment can predispose to IFD, although our understanding of these pathways is incomplete. SARS-CoV-2 infection may be transmitted through contact, droplet, airborne, fomite, faecal–oral, bloodborne, mother-to-child, and animal-to-human routes [25]. Each SARS-CoV-2 virion has an outer surface covered with spike proteins. The S1 subunit hosts the receptor-binding domain (RBD) and is responsible for binding to the human angiotensin-converting enzyme 2 (ACE2) receptor, which is expressed in the lungs and other body sites for cell entry [26,27]. The S2 subunit allows viral fusion with the host cell membrane. Following this, SARS-CoV-2 utilises transmembrane serine protease 2 (TMPRSS2) or cathepsin L to merge the viral and cell membranes. The use of TMPRSS2 pathways leads to more rapid infection [28] as does an intact furin cleavage site within the S protein, as seen in infection with the Alpha and Delta VOC.

The pathological features of COVID-19 are similar to those seen in infection with SARS-CoV and Middle Eastern respiratory syndrome-Coronavirus (MERS-CoV) [5,29], and they are characterised by cell injury and death by pyroptosis [30]. In brief, viral entry and replication leads to the activation of proinflammatory cytokines and chemokines such as interleukin-6 (IL-6), interleukin-8 (IL-8), type II interferon, and monocyte chemoattractant protein 1 [31]. In turn, this leads to the pulmonary recruitment of macrophages and dendritic cells, which are the key components for host innate defences against respiratory infections [32] with the direct viral infection of macrophages and/or dendritic cells [33] or phagocytization of apoptotic-infected cells [34]. These pathways result in further cytokine and chemokine release, whilst the late phase T cell-mediated response is initiated by antigen presentation via dendritic cells and macrophages to promote the production of virus-specific antibody and CD8+ T cells that kill infected alveolar cells. Finally, IL-8, a chemoattractant for neutrophils and T cells, can contribute to lung injury [35,36], with up to 5% of patients experiencing severe lung damage [37].

## 3. COVID-19 Therapies and Risk of IFD 

Therapies used to treat patients with SARS-CoV-2 infection comprise three main categories: (i) antiviral treatments, (ii) immune modulators such as corticosteroids and janus kinase (JAK) inhibitors, and (iii) monoclonal antibody treatments; these can prevent SARS-CoV-2 from entering cells, hence causing serious disease. Immunotherapies are increasingly used, and whilst they block undesired inflammatory effects, they have the potential to increase the risk of IFDs—in particular, corticosteroids such as dexamethasone are routinely used in patients with COVID-19 infection who are receiving oxygen to modulate the systemic inflammatory response [38]. IL-6 inhibitors e.g., tocilizumab, reduce the cascade of cytokine release and JAK inhibitors e.g., baricitinib inhibit cell signalling processes [39]. The currently available drugs including repurposed drugs to treat COVID-19 and their reported associations with IFD are summarized in Table 1. Broadly, immunotherapies increase risk of IFDs through resultant cytopaenias, inhibition of cell signalling, and inhibition of function of T cells, B cells, and/or phagocytes, all of which can lead to increased airway colonization of fungus [40]. Since many of the novel therapies for COVID-19 only have emergency use authorisation rather than full regulatory approval, close monitoring of their use is required to identify adverse consequences including that of subsequent IFD.

## 4. Scope and Pathogens 

### 4.1. COVID-19-Associated Pulmonary Aspergillosis (CAPA)

Much of the literature has focussed on CAPA detailing its burden, clinical features, diagnosis, and outcomes [8,51,52,53,54]. Whilst the present review is directed at non-*Aspergillus* IFDs, it is essential to acknowledge the lessons learned from CAPA, particularly with regard to limitations in diagnosis [23,55]. Other notable challenges with regard to the comparability of data include the definitions used to classify the likelihood of IFD in the setting of COVID-19, study design, and the patient population studied. Currently, the assignment of CAPA cases takes into consideration existing definitions for IA in critical care and influenza-associated pulmonary aspergillosis whilst leveraging the evolving knowledge of CAPA [18,23,55,56]. 

With the above limitations in mind, the estimated incidence of CAPA is reported to be ≈10% (range 3–39%) with an overall mortality of about 50% [53]; many of these patients were in the intensive care unit (ICU). Risk factors include traditional factors for IA such as transplants or haematological malignancies but also those of acute respiratory distress syndrome, mechanical ventilation, corticosteroid use [51,53,55], and possibly, tocilizumab receipt [57]. Consensus expert opinion recommends the use of prospective, multi-modal diagnostic approaches comprising both culture and non-culture-based antigen and nucleic acid amplification tests (NAATs), in conjunction with chest imaging (see Table 2 for the more common radiological abnormalities of CAPA) to be useful in diagnosis. Importantly, the probability of CAPA increases when the positivity of these tests is seen across multiple time points and across multiple sample types [23,55,58]. Hence, it is reasonable to envisage that for other IFDs, similar diagnostic approaches for assigning the probability of infection would apply. Other essential diagnostic tests are pathogen-specific biomarkers where they exist, and histopathological examination for, and culture of pathogens from sterile sites including blood (Table 2).

### 4.2. Invasive Candidiasis

After CAPA, invasive candidiasis (IC) is the second most reported fungal coinfection with COVID-19, [9,10,59,60,61] including infections caused by drug-resistant *Candida* species [60,62]. Risk factors for IC are inevitably present in critically ill COVID-19 patients admitted to the ICU and include mechanical ventilation, indwelling devices, broad-spectrum antibiotic therapy, and glucocorticoid use [63,64]. The incidence of COVID-19-associated candidiasis (CAC) has ranged from 0.7% (7/989) in Spain to 12.6% (17/135) and 23.5% (4/17) in the United Kingdom and China, respectively [63]. Candidemia has predominated amongst the clinical forms of IC with a higher frequency in ICU settings.

Reports suggest there has been a two to 10-fold increase in the incidence of candidemia in patients with COVID-19 with candidemia developing earlier (within two weeks of hospitalisation in the ICU) than in patients without COVID-19 [41,59,65]. The reasons for this are uncertain as differences in underlying comorbidities, disease severity, and classical risk factors for IC between these cohorts have not been identified. Instead, the higher rate may reflect an additive effect of multiple risk factors that may be prolonged and protracted e.g., ICU stay and invasive mechanical ventilation [59,66]. In a large US study of 251 patients with candidemia, 25.5% (64/251) were coinfected with COVID-19 [67]. These patients were less likely to have underlying conditions such as chronic liver disease, solid organ malignancies, as well as traditional risks e.g., prior surgery but more likely to have risk factors linked to treatment for severe COVID-19 that is, tocilizumab and corticosteroids, and mechanical ventilation compared with those without COVID-19; the mortality rate was 62.5% vs. 32.1% [67]. Of note, Kayaaslan et al. demonstrated that corticosteroid treatment was an independent risk factor associated with mortality in patients with candidemia [41]. Other studies have reported mortality rates among patients with *Candida* and COVID-19 of 46–92.5%; the highest rates were in those with *C. glabrata* and *C. auris* infections [41,63,65,68].

*C. albicans* has been the most common pathogenic species 44.1% (19/43), followed by *C. auris* 23.2% (10/43), *C. glabrata*, *C. parapsilosis*, and *C. tropicalis* 4.6% (2/43) each [63]. However, in India, multi-drug-resistant *C. auris* was the most prevalent species [63], and this species has also recently displaced *C. albicans* as the most common *Candida* species in at least one hospital in Spain [69]. It is curious that at the present time of heightened infection prevention measures in hospitals, *C. auris* has emerged as one of the more frequent causative species in hospitals. One hypothesis, yet unproven, is that with enhanced infection control approaches in the COVID-19 setting, paradoxically, those that minimise the exchange of certain practices e.g., keeping on a base layer of gown between patients, may possibly promote the spread of *C. auris* [70].

The diagnosis of IC may be challenging, relying on both conventional culture from blood or other samples, and culture-independent tests including *Candida* mannan and anti-mannan IgG, serum (1,3)-β-D-glucan (BDG), and NAAT-based assays, such as the T2Candida assay [71] (Table 2). The diagnostic sensitivity and specificity may be further increased when serum BDG is combined with procalcitonin to help differentiate fungal from bacterial infections [72]. The high negative predictive value of BDG for diagnosing IC, in the ICU population, may guide the early discontinuation of empiric antifungal therapy if pre-treatment serum BDG is negative [73]. As patient inflammatory responses (e.g., fever) may be blunted following the receipt of immune-modulating agents, a high index of suspicion for IC is required particularly for critically ill COVID-19 patients. Cultures from blood and other sties should be undertaken (Table 2), and empiric anti-*Candida* therapy may be initiated according to institutional protocols.

The treatment of candidemia and other forms of IC is informed by available guidelines, as is addressing modifiable risk factors for source control and to prevent the occurrence of candidemia [68,74]. Echinocandins (anidulafungin, caspofungin, or micafungin) have been the primary antifungals used to treat CAC with liposomal amphotericin B and the azoles (e.g., fluconazole, isavuconazole, posaconazole, and voriconazole) are used as second line alternatives informed by susceptibility test results [64]. As for non-COVID-19 patients with candidemia, the removal of indwelling vascular catheters is essential where possible [63]. Infections due to multi-drug-resistant species, particularly *C. auris*, remain a management challenge.

### 4.3. Pneumocystis Pneumonia 

Uncommon in COVID-19 patients compared with CAPA, *Pneumocystis jirovecii* pneumonia (PCP) coinfection was first diagnosed by real-time PCR at autopsy in a patient with severe dyspnoea [75]. Since then, positive PCR tests for PCP have been increasingly reported in association with COVID-19, including in patients with underlying human immunodeficiency virus (HIV) infection [76,77,78,79,80]. Reported frequencies of positive *P. jirovecii* PCR findings have ranged from 1.4% (2/145) [81] to 9.3% (10/108) [82], but the true incidence of *P. jirovecii* infection as opposed to the detection of *P. jirovecii* DNA in clinical samples which can represent colonisation only is unknown [83]. A review [77] of 12 COVID-19 patients with PCP found all required invasive mechanical ventilation, and many had HIV (58.3%) or were in receipt of long-term immunosuppressive agents such as corticosteroids (91.7%). In the above patients, severe lymphopenia (<1000 cells/mm^3^) was present with a CD4+T cell count of <200 cells/mm^3^ [77], where severe CD4+ lymphopenia is a well-known risk for PCP [80]. It is hypothesised that the development of lymphopenia requiring adjunctive steroids and/or immunomodulatory therapies in COVID-19 patients may re-activate or “activate” asymptomatic *P. jirovecii* infection in colonised patients [82].

The diagnosis of PCP is similar to that in non-COVID-19 populations with combined assessment of clinical features, radiologic findings, and laboratory tests. Diagnosis is challenging because of the similar clinical (e.g., cough, dyspnoea) and radiological presentations of PCP and COVID-19. Undertaking chest computerised tomography (CT) scanning is essential, with extensive diffuse ground glass opacities and interstitial infiltrates being typical, most predominantly in the upper lobes and perihilar regions [76,84]. However, it is important to acknowledge that imaging abnormalities cannot distinguish PCP from COVID-19 pneumonia, and establishing a diagnosis of PCP relies on microbiological approaches. The detection of *P. jirovecii* cysts and/or trophozoites in tissue, bronchoalveolar lavage (BAL) fluid, or expectorated sputum using conventional microscopy or immunofluorescence staining provides a definitive diagnostic of infection but lacks sensitivity, particularly in non-HIV patients [71]. However, the diagnosis of PCP by PCR alone is not sufficiently definitive, as it is unable to discriminate between colonisation and infection; although quantitative PCR (qPCR) allows an estimate of fungal burden, clinical cut-off values have not been established. Nonetheless, a negative qPCR result can rule out PCP. A high fungal load is helpful to establish probable disease, but a lower fungal load detected by qPCR requires additional diagnostic indicators [71,85]. A positive serum BDG (≥80 pg/mL) result can contribute to the diagnosis, particularly when combined with a positive PCR result, and negative BDG results can exclude infection in at-risk patients [71,86]. In COVID-19 patients, the utility of serum BDG to diagnose PCP is particularly appealing as it negates the need to perform invasive procedures [80]. However, more study is required to assess the clinical utility of both qPCR and BDG testing in COVID-19/PCP coinfections. Finally, the occurrence of PCP reminds us of the importance of testing for HIV/AIDS, regardless of COVID-19.

Antifungal therapy should follow a similar approach to patients without COVID-19 [87]. Trimethoprim–sulfamethoxazole often in conjunction with corticosteroids remains the preferred first-line treatment of PCP [87,88], although there is debate as to whether to treat patients coinfected with COVID-19 or not, as some patients have improved without treatment [81,82,83]. When there is a high clinical suspicion for PCP, treatment can be initiated before making a definitive diagnosis, and clinical improvement can be expected within 4–8 days [77]. To date, the use of trimethoprim–sulfamethoxazole has not been associated with adverse outcomes.

### 4.4. Non-Aspergillus Mould Infections

In comparison with CAPA, coinfection or superimposed infections with less common mould pathogens are less frequently reported but should be considered, given the easy access of a myriad of airborne fungi to the respiratory tract and underlying severe COVID-19-induced lung damage. Infections include those due to the Mucorales, *Fusarium*, *Scedosporium/Lomentospora*, and dematiaceous moulds. Where a non-*Aspergillus* mould infection is suspected (e.g., poor response to anti-*Aspergillus* therapy), additional diagnostic evaluation of respiratory tract specimens for these agents is recommended, which may include extended mycological culture and non-culture-based PCR tests [12]. 

A review of the literature indicates that the majority of non-*Aspergillus* mould infections are caused by the Mucorales [89,90,91]. Notably, the number of case reports and small case series of COVID-19-associated mucormycosis (CAM) has increased substantially with the rise of infection with COVID-19 in India in mid 2021 with many infections developing in patients recovering from COVID-19 [92,93,94,95]. Only one case each of invasive fusariosis and mixed mould infection are reported [96,97] and as of yet, there are no reports of fungemia caused by mould pathogens.

Risk factors for the acquisition of non-*Aspergillus* mould infections are similar to those for CAPA. In addition, for CAM, poorly controlled diabetes mellitus and trauma, in addition to underlying haematologic malignancy and allogeneic haematopoietic stem-cell transplantation (HSCT), have comprised the majority of co-morbidities; all are well-established risks for these infections [98]. In patients with CAM without traditional risk factors, many had hypertension, had end-stage kidney disease, and had received corticosteroid treatment for COVID-19. A recent review of 41 cases of CAM showed that underlying diabetes was present in 94% of cases and associated with severe COVID-19 in 95% cases [99], as did that of Selarka et al. of 47 patients across three centres (76.6%, 91.55%) [100]. As expected, corticosteroid use was a key risk for mucormycosis from resultant hyperglycaemia [101]. Furthermore, the high expression of ACE-2 receptors in pancreatic isolates with resultant insulin resistance may predispose to diabetes. The hyper-ferritinemic state and intracellular iron load of severe COVID-19 as well as the presence of endothelialitis poses risk for mucormycosis, as summarized in John et al. [99]. Contaminated medical supplies, equipment, and environmental factors have been suggested as risk factors for community and nosocomial CAM in India [102].

Case reports have identified that mucormycosis usually develops 10–14 days after hospitalisation and in some cases was detected only at *postmortem*. Clinical presentation comprises mostly rhino-orbital/rhino-orbital cerebral (ROCM) disease, which is typical of that seen in patients with diabetes mellitus. In contrast to CAPA, nearly all CAM infections have been classed as proven infections [99]. As invasive mucormycosis and fusariosis both share many common features with CAPA in critically ill COVID-19 patients, clinical vigilance is paramount in recognising these serious mimickers of CAPA where even in survivors, morbidity is high including loss of vision; in-hospital mortality was 49% [99]. The absence of reports of coinfections with the *Scedosporium*/*Lomentospora* may reflect limitations in diagnostics. These pathogens are important in hospital epidemiology and should be considered in all vulnerable immunocompromised patients such as those with haematological malignancy, stem cell, and solid organ transplantation [103,104].

The diagnosis of non-*Aspergillus* mould infections follows similar principles to those for other IFDs [98,105,106]. Clinical suspicion should prompt appropriate imaging and examination of clinical specimens (sputum, tracheal aspirates, BAL fluid, skin lesions) by histology, direct microscopy, culture for fungi, and employment of antigen and NAAT-based approaches. *Fusarium* and *Scedosporium*/*Lomentospora* may be isolated from blood cultures. On histological examination and direct microscopy, Mucorales demonstrate broad pauci-septate irregular hyphae, whilst other non-*Aspergillus* moulds appear more slender. The invasion of blood vessels and tissue can be seen with Grocott–Gomori’s methenamine silver (GMS), haemotoxylin and eosin (H&E) and Periodic-Acid Schiff (PAS) stains [98,107]. Identification of the pathogen by Matrix-Assisted Laser Desorption Ionization—Time of Flight Mass Spectrometry (MALDI-TOF MS) systems and DNA sequencing targeting the internal transcribed spacer region (ITS) or large ribosomal subunit may be necessary for species identification. Panfungal PCR may be attempted on tissue specimens to directly detect the pathogen [98,105]. 

**Figure 1:**
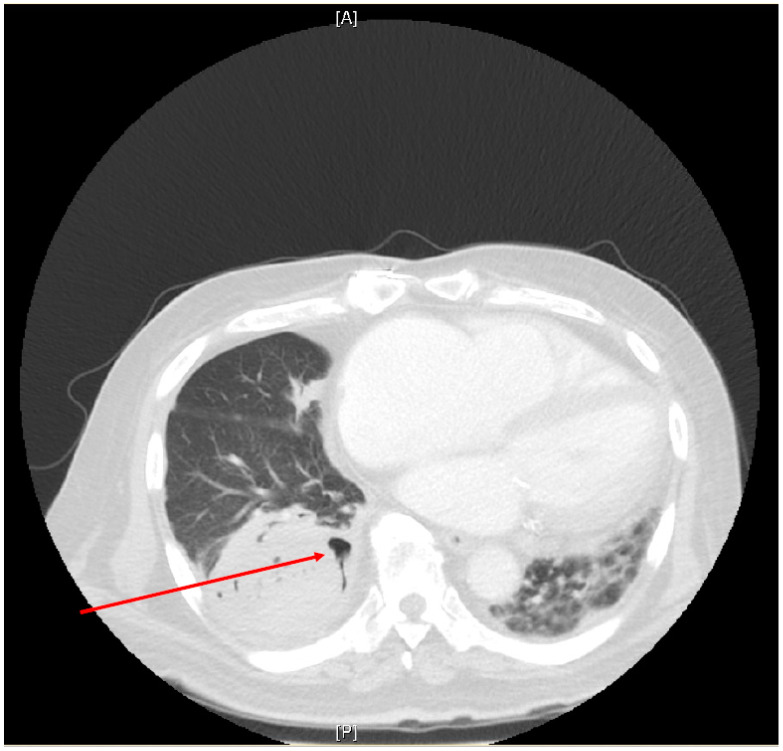
Chest Computer Tomography (CT) scan of a patient who had recovered from COVID-19 two months prior to presenting with new onset right-sided lower chest pain. Abnormalities on CT scan include a large mass lesion in the postero-basal segment of the right lower lobe with the beginnings of possible cavitation (arrow). A pleural effusion is present at the right lung base. The left lung shows consolidation with ground glass opacities. Fine needle aspiration of the right lower lobe mass yielded *Rhizopus microsporus* on culture with broad, pauci-septate irregular fungal hyphae seen on standard histopathological stains. [A]—anterior; [P]—posterior.

Approaches to imaging are dependent on the site of involvement; in cases of suspected CAM where there is lung involvement, it is expected that abnormalities typical of mucormycosis in other patient groups will be present (Table 2). On chest CT, these include ground glass opacities and mass lesions with or without cavitation and consolidation (see Figure 1, which shows some of these abnormal features). The reverse halo sign, considered to be pathognomonic of pulmonary mucormycosis, may or may not be evident. Vascular occlusion on chest angiography may be present. Detailed descriptions are beyond the scope of this review but can be found in a recent international guideline of managing mucormycosis [98]. In addition, where CAM is suspected, imaging of the sinuses and brain is essential as well as of other body sites as clinically indicated [98]. Imaging abnormalities in the lung for other mould infections may be expected to mimic both CAM and CAPA [105] (Table 2). 

Similarly, the principles of prompt antifungal therapy and early surgical debridement apply as for other patients with these infections [98,105]; however, surgery may not be feasible in COVID-19 patients for the same reasons why performing diagnostic procedures such as bronchoscopy may be problematic. There are no data on the impact on patient outcomes of specific therapies in the setting of COVID-19. For Mucorales infections, a lipid amphotericin B formulation, typically liposomal amphotericin B, is recommended as first-line monotherapy, with isavuconazole and posaconazole being alternatives; both azoles may be used as salvage therapy. There is no evidence to indicate the survival benefits of either therapy with polyene-azole or polyene-echinocandin combinations. For invasive fusariosis, primary treatment with either voriconazole or a lipid amphotericin B formulation is appropriate; however, combination therapy with these two agents is frequently used because of the presence of severe disease and due to challenges in achieving therapeutic voriconazole levels. For the treatment of other uncommon mould infections, the reader is referred to the management recommendations by Hoenigl et al. [105]. 

### 4.5. Endemic Mycoses

Particularly in geographic regions where endemic mycoses occur, lung infection caused by agents of these mycoses may coexist with COVID-19. As for CAPA and other mould infections, diagnosis may be missed given the similarity of presentation. Patients with severe COVID-19 and/or those receiving significant immunosuppressive therapy may experience reactivation of dormant or past infection with an endemic fungus. The wide use of corticosteroids, specifically dexamethasone, or IL-6 inhibitors, and other immunosuppressants to treat severe COVID-19 increase the risk of symptomatic endemic mycoses, as summarised in Segrelles-calvo et al. [20].

At least three patients with *Coccidioides* and SARS-CoV-2 coinfection have been reported [108,109,110]. Infections may be subclinical and diagnosed during the recovery phase of COVID-19. Heaney et al. [111] have described possible social, demographic, and exposure risk factor interactions between coccidioidomycosis and COVID-19, focusing on racial and ethnic minorities and the role of geography [111]. In addition, chronic lung disease from coccidioidomycosis may increase the risk of severe COVID-19, and COVID-19 may increase the risk of reactivation of latent *Coccidioides* infection [111].

Four coinfections with *Histoplasma* and SARS-CoV-2 have been reported, all from South America [112,113,114,115], with three cases occurring in the context of HIV infection. As with PCP, opportunistic infections typically associated with HIV should be considered in the differential diagnosis in patients with suggestive radiological features even if they have received a COVID-19 diagnosis. The coinfection reported in a HIV-negative patient was in the setting of persistent pulmonary histoplasmosis when pulmonary imaging prompted SARS-CoV-2 testing [115]. Typical radiological abnormalities for the endemic mycoses are summarised in (Table 2) [116]. SARS-CoV-2 coinfections with other dimorphic fungi—*Blastomyces*, *Emergomyces*, *Paracoccidioides*, *Sporothrix*, or *Talaromyces*—have not yet been reported but would not be unexpected in the appropriate clinical context.

Diagnostic approaches should consider culture, microscopy, serologic antibody, and antigen tests on blood or urine and NAAT as appropriate. In most patients, diagnostic testing for coccidioidomycosis typically begins with antibody tests, with enzyme immunoassay (EIA) being most widely available, and less commonly antigen testing [117]. Antibody testing can also be used but is less reliable for histoplasmosis [118] and blastomycosis [119]. Treatment guidelines for endemic mycoses from the Infectious Diseases Society of America (IDSA) recommend that mild or moderate illness can be treated with triazole antifungals and severe disease can be treated with amphotericin B preparations followed by triazoles [119,120,121]. 

### 4.6. Cryptococcosis

Coinfection of SARS-CoV-2 and *Cryptococcus* has been uncommonly reported but may reflect under-recognition. Clinical manifestations include cryptococcemia, lung infection, and meningoencephalitis [122,123,124]; infection may also be disseminated [125,126]. Cryptococcosis may occur concurrently or present after recovery from COVID-19 and even diagnosed post mortem [124]. Patients had underlying immunocompromise and/or had received corticosteroid therapy for COVID-19, but cryptococcosis may also affect apparently immunocompetent hosts [123]. Imaging abnormalities in the lung in non-COVID-19 patients range from small nodules to large cryptococcomas or mass lesions, or non-specific pulmonary infiltrates [127]; whether these features will be borne out in the COVID-19 setting is uncertain. Imaging of other body sites should be guided by clinical decisions. 

Laboratory-based diagnostic approaches are similar to those in non-COVID-19 patients, with histopathology, culture, and cryptococcal antigen tests being the cornerstone supported by molecular assays. In cases of suspected meningitis, a lumbar puncture should always be performed and cerebrospinal fluid examined by microscopy and culture [128]. Antifungal treatment and management of complications e.g., raised intracranial pressure, would be guided by recommendations of existing guidelines such as the IDSA [128]. A single patient has received isavuconazole therapy, rather than fluconazole, following induction therapy with liposomal amphotericin B [122]; there are sparse data on the role of this azole in treating patients with cryptococcosis.

## 5. Approach to Diagnosing a Suspected Fungal Co-Infection in COVID-19 Patients 

The first step in managing any COVID-19-associated IFD is to identify high-risk patients and evaluate them for IFD. Broadly, patients with severe COVID-19 who require mechanical ventilation are at risk for CAPA [23,53], other mould infections, and PCP. In addition, non-ventilated COVID-19 patients in the ICU who are experiencing clinical deterioration (e.g., pulmonary desaturation, sepsis-like syndrome) with no identifiable cause should also be evaluated for IFD, including for candidemia [129]. Additional risks such as diabetes mellitus in CAM and predisposing factors for IC are noted in their respective subsections above. Travel to an endemic region or prior infection could be a risk for infection or reactivation with an endemic mycosis during or after COVID-19 illness (Table 2). Whilst these factors may offer diagnostic clues, they cannot replace the importance of carefully working through the patient history and embarking on appropriate diagnostic work-up. 

Diagnostic approaches are similar to those in non-COVID-19 patients, encompassing laboratory-based methods including histopathology, culture-based, serology, and NAAT -based methods, often in combination along with radiology to optimise the diagnostic yield. Table 2 summarises recommendations for the use of various methods to assist with the diagnosis of the major groups of fungal infections and includes CAPA for comparison. As described in the sections above, the histopathological examination of tissue specimens especially is strongly recommended where practicable, and culture remains a cornerstone for the diagnosis of many of these IFDs. As the agents of endemic mycoses are risk group 3 pathogens, the culture of specimens in suspected cases must be performed in a physical containment level 3 laboratory if attempted. Serological techniques remain useful in the diagnosis of cryptococcosis and for certain endemic mycoses (e.g., histoplasmosis). The increasing availability of NAAT assays for a large range of fungi has good potential to enable rapid accurate diagnosis either through panfungal or genus/species-specific approaches. Finally, the major imaging abnormalities that can be anticipated to be present in each group of IFD are summarised.

## 6. Conclusions

As COVID-19 continues to spread worldwide, the emergence of VOC with vaccine escape potential, slow and uneven global uptake of vaccinations, and waning immunity in individuals vaccinated greater than 6 months ago has resulted in a largely susceptible population globally, making it important to continue to consider coinfections including fungi especially in the at-risk groups highlighted here in this review. Non-*Aspergillus* infections are a broad group of IFDs that may occur during or after the episode of COVID-19. Diagnostic algorithms are currently largely based on standard of care approaches practiced pre COVID-19, informed by guidelines, and encompassing multi-modal approaches. These will need to be updated as new learnings emerge from our growing experience of these infections in the setting of COVID-19. Antifungal and other management strategies are similar to those in non-COVID-19 patients but may be tempered by coincident comorbidities, accompanying drug use, and a whole patient approach. 

## Figures and Tables

**Table 1:** Summary of therapies used for COVID-19.

Drug Category	Drugs	Mechanism of Action	Fungal Infections Reported
Antiviral drugs	remdesivir, (Veklury^®^, Gilead Sciences Inc.)	Nucleoside anti-proviral drug Inhibits SARS-CoV-2 replication via RNA-dependent RNA polymerase (RdRp)	Nil reported
Immune modulators	Glucocorticoids e.g., predinisone prednisolone dexamethasone	Decrease vasodilation, permeability of capillaries, and leukocyte migration Inhibit neutrophil apoptosis and demargination; inhibit phospholipase A_2_ function, and inhibit NF-Kappa B and inflammatory transcription factors Promote expression of anti-inflammatory genes such as that for IL-10.	Candidiasis Pneumocystosis Invasive aspergillosis Mucormycosis [41,42]
	baricitinib, (Olumiant^®^, Eli Lilly and Company)	JAK inhibitors: Bind to JAK, which prevents the activation of the JAK–STAT signalling pathway, which reduces the production of proinflammatory cytokines	Candidiasis Pneumocystosis Histoplasmosis Cryptococcosis ** [43,44]
	tofacitinib, (Xeljanz^®^, Pfizer)		Oesophageal candidiasis Cryptococcosis [45,46,47]
Monoclonal antibodies (mAb)	tocilizumab, (Actemra^®^, Roche)	IL-6 receptor antagonist. Results in reduction in cytokine and acute phase reactant production.	Invasive candidiasis Cryptococcosis Pneumocystosis [47,48,49]
	sotrovimab, (Xevudy^®^, GlaxoSmithKline)	Engineered human IgG1 monoclonal antibody that binds to the spike protein receptor binding domain (RBD) of SARS-CoV-2	Nil reported to date
	sarilumab, (Kevzara^®,^ Sanofi and Regeneron Pharmaceuticals, Inc.)	IL-6 receptor antagonist. Results in reduction in cytokine and acute phase reactant production.	Candidiasis Pneumocystis. ** [50]
	casirivimab and imdevimab (REGEN-COV™, Regeneron Pharmaceuticals, Inc.)	Casirivimab (IgG1κ) and imdevimab (IgG1λ) bamlanivimab (IgG1κ) and etesevimab (IgG1κ) Recombinant human monoclonal antibodies that bind to the spike protein RBD of SARS-CoV-2, which leads to the blocking of binding to the human ACE2 receptor, thereby preventing viral attachment to host cells	Nil reported to date
	bamlanivimab and etesevimab (Eli Lilly and Company)		

Key: ACE2—angiotensin-converting enzyme 2; IL-6, interleukin-6; IL-10, interleukin-10; JAK—janus kinase; STAT—signal transducers and activators of transcription; ** patients with invasive fungal infections may present with disseminated rather than localised disease.

**Table 2:** Summary of risk factors in COVID-19 patients for invasive fungal disease and diagnostic approaches.

		CAPA	IC	PCP	Cryptococcosis	Endemic Mycoses	Non *Aspergillius* Mould Infections	CAM
RISK FACTORS								
Corticosteroid receipt		X	X	X	X	X	X	X
ICU MV or non MV + (clinical deterioration)		X	X	X	X	X	X	X
IL-6 inhibitor therapy		X	X			X		
HIV/severe lymphopenia				X	X			
Receipt of immunosuppressive therapies		X	X	X	X	X	X	X
Poorly controlled diabetes mellitus								X
Major trauma							X	X
Travel to endemic region or previous infection with endemic mycoses						X		
DIAGNOSTIC APPROACHES								
Histopathology	Characteristics findings using standard stains	•	•	•	•	•	•	•
		Hyaline, acutely branching septate hyphae	Budding yeast cells and/or pseudohyphae	Cysts and/or trophozoites	Encapsulated yeast cells	Budding yeast cells or spherules	Hyaline, branching septate hyphae	Broad, irregular, pauci septate hyphae
Culture-based	Characteristic findings on microscopy	•	•	•	•	•	•	•
		Hyaline, acutely branching septate hyphae	Budding yeast cells and/or pseudohyphae	Cysts and/or trophozoites	Encapsulated yeast cells	Budding yeast cells or spherules	Hyaline, branching septate hyphae	Broad, irregular, pauci septate hyphae
	Respiratory tract	•	•		•	•	•	•
	Sterile sites other than blood				•		•	•
	Blood		•		•		•	
Non-culture-based	*Aspergillus* Ag	•						
	Cryptococcal Ag				•			
	EIA for antibody detection or Ag testing for *Coccidioides* and/or *Histoplasma*					•		
	Serum 1,3, β-D-glucan	•	•	•			•	
	Genus-specific NAAT	•	•	• **^^**	•	•	•	•
	Panfungal PCR (ITS1/2)	•	•	•	•	•	•	•
RADIOLOGY (typical or more common abnormalities on chest CT)		Peripheral, bilateral GGO +/− consolidation or visible intralobular lines (i.e., crazy paving) in early stages. Multifocal GGO (round) +/− consolidation or intralobular lines at peak stage. Reverse halo sign +/− organising pneumonia at late stage **	As directed by clinical findings; organ involvement rare	Diffuse GGO Interstitial infiltrates (predominantly upper lobes and perihilar regions)	Nodules (1 or more) Cryptococcomas, Pulmonary infiltrates	Focal or diffuse airspace disease Upper lobe cavitation thick-walled bullae, lymphadenopathy	Similar to CAM and CAPA	GGO, mass lesions +/− cavitation Consolidation, Reverse halo sign may be present. ##

Key: Ag—antigen; CAPA—COVID-19-associated pulmonary aspergillosis; CAM—COVID-19-associated mucormycosis; CT—computerised tomography; EIA—enzyme immunoassay; GGO—ground glass opacities; HIV—human immunodeficiency virus; IC—invasive candidiasis; ICU MV or non MV—intensive care unit admission with mechanical ventilation or without mechanical deterioration + clinical deterioration (e.g., pulmonary desaturation, sepsis-like syndrome); IL-6 (interleukin 6, i.e., Tocilizumab, and Sarilumab); ITS—internal transcribed spacer region; NAAT—nucleic-acid amplification test; PCP—*Pneumocystis jirovecii* pneumonia; X—denotes the presence of the risk factor well established to be associated with the fungal infection; •—denotes a recommended test; **^^**—quantitative PCR; ** Findings can be atypical; lobular or segmental consolidation in predominantly cavitating, tree in bud opacities with peri hilar nodules; ##—in suspected CAM—other sites including the sinuses and brain must also be imaged in addition to the chest.

## Data Availability

Not applicable.
